# Isolated primary diffuse large B-cell lymphoma in thumb, the first case in medical literature: Case report and literature review

**DOI:** 10.1097/MD.0000000000043959

**Published:** 2025-08-22

**Authors:** Khder Yousf, Ahmad Osman, Mkdad Dalla, Loujain Zaher, Tala Zaher, Rabab Salloum, Firas Hussein

**Affiliations:** a Faculty of Medicine, Tishreen University, Lattakia, Syria; b Cancer Research Center, Tishreen University, Lattakia, Syria; c Faculty of Medicine, Al Andalus University, Tartous, Syria; d Department of Pathology, Tishreen University Hospital, Lattakia, Syria; e Department of Hematology, Tishreen University Hospital, Lattakia, Syria.

**Keywords:** DLBCL, PBL, thumb

## Abstract

**Rationale:**

Primary lymphoma of the bone is a rare lymphoma of bone origin. Diffuse large B-cell lymphoma (DLBCL) is the most common type of cancer worldwide. The femur is the most abundant site, followed by the pelvis, vertebrae, and humerus, and may affect one or several bones simultaneously.

**Patient concerns:**

A 60-year-old female visited the hospital with a history of pain and swelling in her left thumb, without a history of trauma or other symptoms.

**Diagnoses:**

After conducting these tests, we diagnosed her with isolated DLBCL in the thumb.

**Interventions:**

We treated her according to the R-CHOP protocol with a combination of rituximab, vincristine, endoxan, doxorubicin, and prednisone for 4 cycles.

**Outcomes:**

After completing the treatment, the patient achieved a complete recovery, and this result is supported by the results of normal tests and the patient’s normal positron emission tomography scan.

**Lessons:**

Isolated thumb involvement in DLBCL is rare, and to the best of our knowledge, no similar case has been previously reported. Therefore, it is important to consider DLBCL during the differential diagnosis of similar cases.

## 1. Introduction

Primary bone lymphoma (PBL) is a rare lymphoma of bone origin, which constitutes <1% of all malignant lymphomas, 7% of all bone cancers and 4% to 5% of lymphomas that develop outside the lymph nodes, noting that diffuse large B-cell lymphomas (DLBCL) is the most common type in PBL.^[1]^ A slight predominance of males has been documented, and it can occur at any age, with a median age of 48.^[2]^ The most common location of PBL is usually in the femur, then the pelvis, vertebrae and humerus, usually in the metadiaphyseal region of the bone and about 10% to 40% of cases are multifocal, causing multiple lesions in 1 bone or involving several bones at the same time (polyostosis).^[3]^ The percentage of DLBCS presenting as a limited low-grade tumor is about a quarter to a third of all cases.^[4]^

In this article, we discuss the diagnosis and treatment of a rare case of primary bone diffuse large B-cell lymphoma (PBDLBCL) of the thumb.

## 2. Case presentation

**Figure 1.**
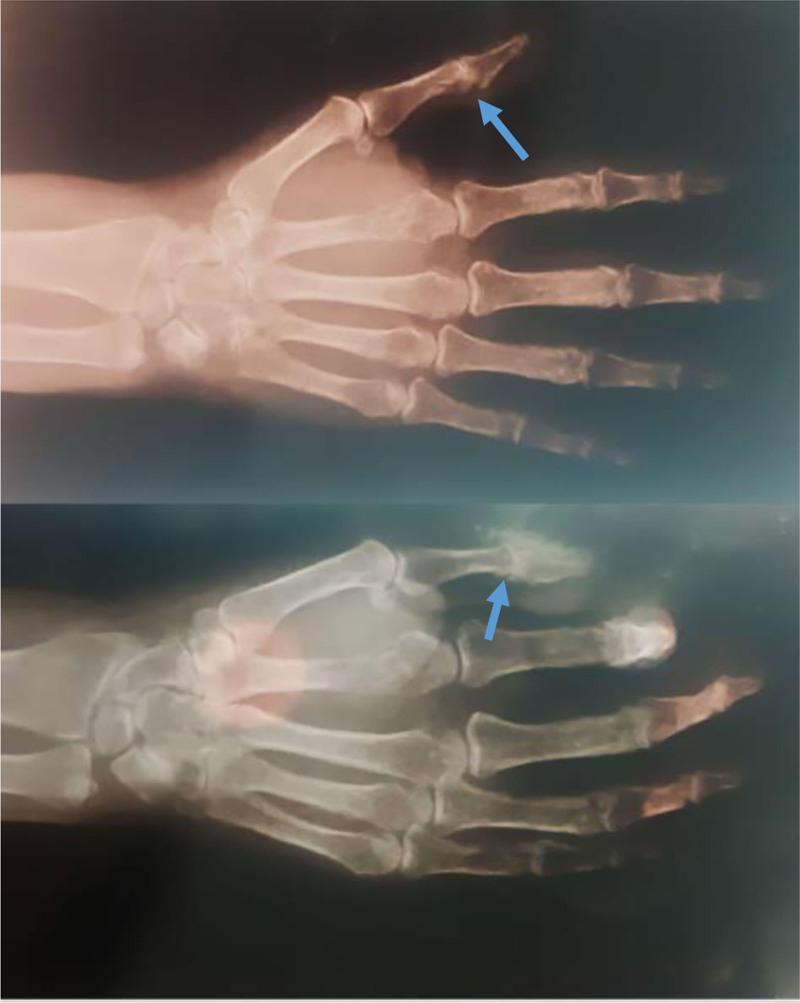
X-ray showing an osteolytic lesion involving the distal part of the first phalanx and the proximal part of the second phalanx of the thumb.

A 60-year-old female came to our hospital clinic complaining of pain and swelling in her left thumb without a story of trauma. An X-ray examination revealed osteolytic lesions involving the distal part of the first phalanx and the proximal part of the second phalanx of the thumb, including the joint (Fig. 1). The patient was admitted to the hospital for evaluation and diagnosis.

In the patient’s history, the patient is a nonsmoker, nonalcoholic and suffers from type 2 diabetes, hypertension, and hypothyroidism, all of which are well controlled with medications. A physical examination was performed, which revealed a slight limitation of movement of the interphalangeal joint of the thumb. There were no signs of lymphadenopathy or any other accompanying disorders.

Hematology workup showed an increase in erythrocyte sedimentation rate (Table 1).

**Table 1:** Hematology workup results.

Leucocytes	10.4 × 10^3^/mm^3^
Neutrophils	69.4%
Lymphocytes	26.6%
Erythrocytes	4.21 × 10^6^/mm^3^
Hemoglobine	12 g/dL
Hematocrite	37.80%
Mean corpuscular volume (MCV)	89.79 fl
Mean corpuscular hemoglobin (MCH)	28.5 pg
Mean corpuscular hemoglobin concentration (MCHC)	31.75 g/dL
ESR	(1 h) 37 mm
	(2 h) 75 mm
Platelets	161 × 10^3^/μL

ESR = erythrocyte sedimentation rate.

Serum protein electrophoresis and tumor markers were ordered, and the results were normal.

A full-body CT scan was performed, and no evidence of any lesion was found. As a result, the decision was made to perform a biopsy of the bone lesion.

The histopathology study showed large lymphocytes with moderately abundant basophilic cytoplasm. The nuclei were round to oval with a single prominent central nucleolus. Numerous atypical mitotic figures were noted.

**Figure 2.**
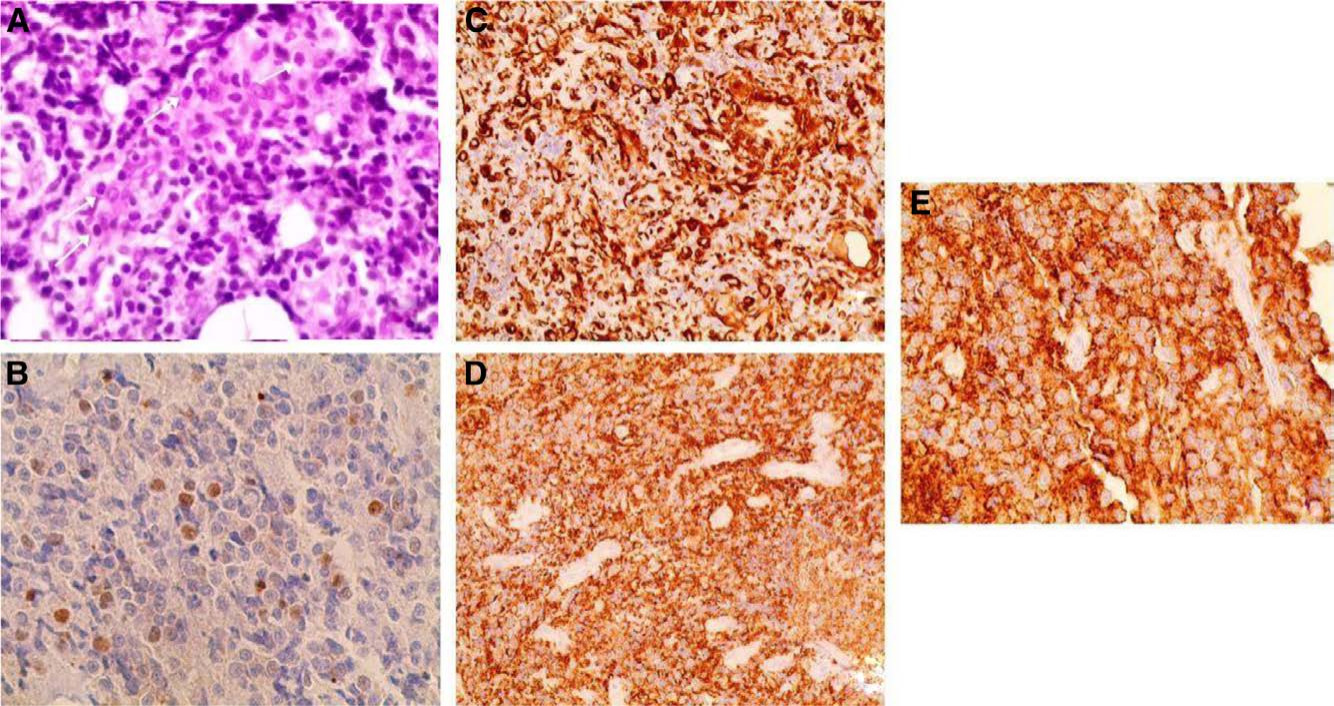
Histological study of osteolytic lesion. (A) Hematoxylin–eosin stain showing large lymphocytes with moderately abundant basophilic cytoplasm and nuclei are circular to oval with a single prominent central nucleolus. (B) Mitotic index (Ki-67) = 40% of tumor cells. (C) Positive staining with vimentin. (D) Positive staining with CD20. (E) Positive staining with LCA. LCA = leukocyte common antigen.

Immunohistochemistry was positive for vimentin, leukocyte common antigen (LCA), and CD20, with high mitotic activity in about 40% of tumor cells (Ki-67 = 40%) (Fig. 2).

The remaining immunostains were negative (CD56, CD99, CD3, CK and chromogranin A).

**Figure 3.**
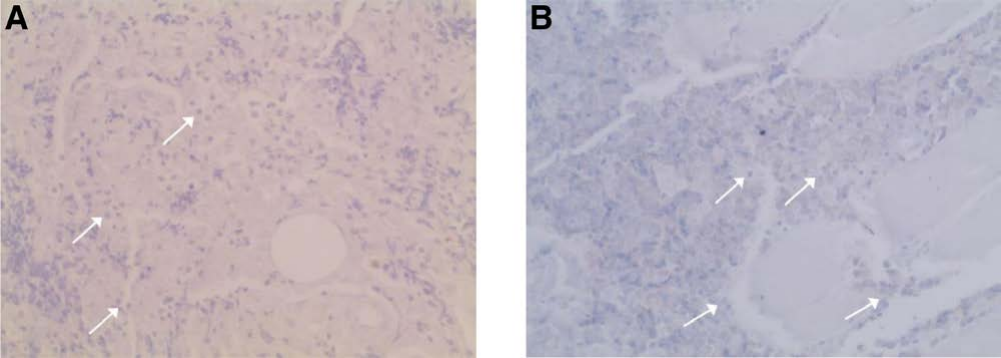
CD20 and Bcl6 immunostaining. (A) Negative staining with CD20. (B) Negative staining with Bcl6.

Further, CD10 and Bcl6 immunostaining were performed and the result was negative, thus the lymphoma was non-follicular (Fig. 3).

From there, the diagnosis was made of non-Hodgkin lymphoma, DLBCL of bone tissue with mitotic index in about 40% of tumor cells, without any signs of external spread.

The patient was treated using the R-CHOP protocol with a combination of rituximab, vincristine, endoxan, doxorubicin, and prednisone for 4 cycles.

After completing the full course of treatment, all laboratory values were normal with LDH value 218 U/L, and PET-scan was ordered, which was completely normal to reflect the patient’s complete recovery.

## 3. Discussion

Non-Hodgkin lymphoma is classified as the most common hematological tumor in the world, which includes a proliferative disorder that affects B-cells and T-cells. It includes more than 40 types, the most common of which are indolent follicular lymphoma and DLBCL.^[5]^ Many studies indicate that PBL constitutes <5% of all extra-nodal lymphomas, <1% of non-Hodgkin lymphomas, and <3% of all bone malignancies.^[6]^ DLBCL is the most common type of non-Hodgkin lymphoma causing PBL.^[7,8]^ DLBCL location includes the tibia, head, neck, and vertebrae, but the metaphysis of the femur is the location that is most frequently involved, noting that DLBCL being located in the hand and fingers is an uncommon condition.^[9]^ As far as we know this case represents the fourth case in the medical literature of PBDLBCL localized in the fingers without rheumatoid arthritis or the use of methotrexate.^[9–11]^ To be precise, according to our knowledge, PBDLBCL in the thumb without being accompanied by rheumatoid arthritis was recorded once, and it was associated with cancer in the femur, which makes this case the first case recorded in the medical literature of isolated DLBCL affecting the thumb.^[12]^ Speaking of the finger joints, this case is the first case recorded of PBDLBCL in the interphalangeal joint in the thumb, indicating that there has been a case reported in the medical literature about injury to the metacarpophalangeal joint of the ring finger in the context of treatment with methotrexate.^[13]^ All of the above-mentioned research we conducted in the medical literature indicates the importance of the case we present in terms of the type of lymphoma and its location. Localized bone pain in the impacted region is the most prevalent sign of PBL, but on the other hand we find that the soft tissue edema, palpable masses, pathological fractures, reduced range of motion in the affected articulation and common symptoms like fever, sweats at night and inadvertent weight loss are other less frequent sings, however accurate diagnosis of PBL is made using a combination of imaging techniques and clinical examinations while histopathological analysis using immunohistochemistry staining is used to confirm the diagnosis.^[14–16]^ In our patient, pain and edema at the site of osteolysis were the main symptoms, without any other symptoms. Pathologically, we find infiltration of cells in the bone and adipose tissue with destruction of the tissue structure, where we find clusters of medium to large-sized cells that are single or multi-lobulated, with condensation of chromatin inside the nuclei and a decrease in the amount of cytoplasm.^[1]^ Immunohistochemical staining in PBDLBCL is essential and reflects positivity of staining for B-cell antigens such as cluster of differentiation 19 (CD19), CD20, LCA/CD45, CD79a and PAX5, and negative of staining for T-cell antigens such as CD3 and CD5.^[14]^ Vimentin is also commonly used in immunohistochemistry in tumors of mesenchymal and melanocytic origin, in addition to some types of epithelial tissue tumors.^[17]^ In our case, we were able to confirm the histological diagnosis through the shape of the cells, their nuclei, their positivity for immunological stainings for vimentin, LCA, and CD20, with high mitotic activity in about 40% of tumor cells, and the negativity of immunostainings for CD56, CD99, CD3, CK, and chromogranin A. Local DLBCL is typically treated with the anti-CD20 monoclonal antibody rituximab and anthracyline-based chemotherapy, but the standard immunochemotherapy is R-CHOP (cyclophosphamide, doxorubicin, vincristine, and prednisone in addition to rituximab).^[18]^ R-CHOP has improved overall survival compared to chemotherapy administered alone.^[19]^ Several randomized clinical trials have demonstrated that 4 cycles of R-CHOP are equivalent to 6 cycles in patient with early stage disease.^[19,20]^ Accordingly, we decided to place the patient on the R-CHOP protocol for 4 cycles; 90% of patients with limited DLBCL and only 60% with advanced DLBCL have been proven to be cured with R-CHOP.^[21]^ In general, PBL has a good prognosis; in fact, it is thought to have the greatest prognosis of all primary malignant bone tumors, even better than that of secondary bone lymphoma.^[14]^

## 4. Conclusion

We report a case of a very rare localization of PBDLBCL. To our knowledge, isolated localization of DLPCL in the thumb without any symptoms or lymphadenopathy is the first case reported in the medical literature and we were able to achieve a complete recovery for the patient by following the R-CHOP protocol with 4 cycles. Therefore, attention should be drawn to this characteristic presentation in the location and symptoms of DLBCL and taken into consideration in the differential diagnosis of such cases.

## Author contributions

**Conceptualization:** Khder Yousf.

**Investigation:** Ahmad Osman, Mkdad Dalla, Loujain Zaher, Tala Zaher.

**Methodology:** Rabab Salloum.

**Resources:** Ahmad Osman, Mkdad Dalla, Tala Zaher.

**Supervision:** Rabab Salloum, Firas Hussein.

**Validation:** Loujain Zaher.

**Writing – original draft:** Khder Yousf.

**Writing – review & editing:** Khder Yousf.
